# Manipulating word awareness dissociates feed-forward from feedback models of language-perception interactions

**DOI:** 10.1093/nc/niv003

**Published:** 2015-07-03

**Authors:** Jolien C. Francken, Erik L. Meijs, Odile M. Ridderinkhof, Peter Hagoort, Floris P. de Lange, Simon van Gaal

**Affiliations:** ^1^ Donders Institute for Brain, Cognition and Behavior, Radboud University Nijmegen, PO Box 9101, 6500 HB Nijmegen, Netherlands;; ^2^ Department of Psychology, University of Amsterdam, Weesperplein 4, 1018 XA Amsterdam, Netherlands;; ^3^ Max Planck Institute for Psycholinguistics, Wundtlaan 1, 6525 XD Nijmegen, Netherlands

**Keywords:** visual perception, language, feed-forward processing

## Abstract

Previous studies suggest that linguistic material can modulate visual perception, but it is unclear at which level of processing these interactions occur. Here we aim to dissociate between two competing models of language–perception interactions: a feed-forward and a feedback model. We capitalized on the fact that the models make different predictions on the role of feedback. We presented unmasked (aware) or masked (unaware) words implying motion (e.g. “rise,” “fall”), directly preceding an upward or downward visual motion stimulus. Crucially, masking leaves intact feed-forward information processing from low- to high-level regions, whereas it abolishes subsequent feedback. Under this condition, participants remained faster and more accurate when the direction implied by the motion word was congruent with the direction of the visual motion stimulus. This suggests that language–perception interactions are driven by the feed-forward convergence of linguistic and perceptual information at higher-level conceptual and decision stages.

## Introduction

**Figure 1:**
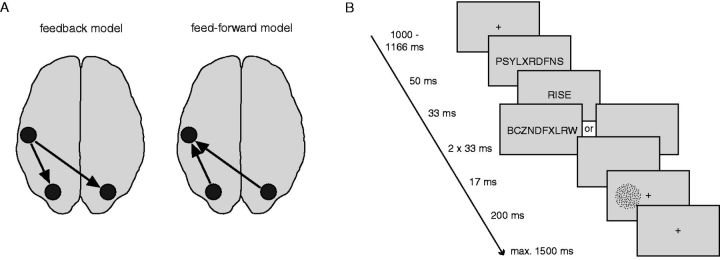
Models and task design (**A**) In the feedback model of language-perception interactions (left), linguistic information is processed in language-specific regions and subsequently feeds back to the sensory system to modulate perceptual processing. Therefore, the processing of visual stimuli is influenced at the level of visual cortex. In the feed-forward model of language–perception interactions (right), linguistic information is likewise processed in language-specific regions where it activates a conceptual representation. Crucially, in this case, the visual information is also processed up to a conceptual level, and it is here at the conceptual level that linguistic information interacts with visual stimuli. (**B**) A congruent or incongruent motion word (upward or downward, e.g. “rise” or “fall“) is displayed in advance of every motion discrimination trial. All words are preceded by a forward mask; unaware words are additionally followed by two backward masks. The visual motion stimulus is presented either in the left or right lower visual field and the dots move upward or downward.

A growing body of evidence shows that language affects perception (e.g. Meteyard *et al.*, 2007; Winawer *et al*., 2007; Thierry *et al.*, 2009; Landau *et al.*, 2010; Lupyan, 2012). However, it is unclear whether linguistic material changes information processing at low-level sensory stages (perceptual level) or whether these “language-perception interactions” are mediated by effects at higher cognitive levels of representation (conceptual level) or even at later perceptual decision stages. Here, we refer to perception as encompassing both the raw sensory processing of a visual stimulus as well as the transformation of this event into a categorical decision. In this study, we aim to dissociate between two models that favor low-level versus higher-level interactions, respectively. In the first model, which we call the “feedback model” (Fig. 1A), linguistic information is processed in language-specific regions and then feeds back, or is “broadcasted” to lower-level sensory regions to modulate perceptual information processing. For instance, the activation of the semantic representation of the motion-implying word “rise” in the temporal cortex may feedback and affect the sensory representation or processing of visual motion stimuli (i.e. moving dots) in hMT+/V5. This feedback model is one of the dominant views in the field (Meteyard *et al*., 2007; Lupyan, 2012). In line with this model, and the view that language comprehension reflects an “embodied process” (Barsalou, 2008), words or sentences describing motion have been shown to activate motion-sensitive visual areas that process actual visual motion (Saygin *et al.*, 2010). Similarly, predictive processing theories have proposed that motion words may induce an “automatic top-down prediction” about visual motion, thereby automatically recruiting hMT+/V5, in a way that is similar to how expectation affects visual perception (Hirschfeld and Zwitserlood, 2011; Lupyan, 2012; Summerfield and de Lange, 2014). However, these theories are yet to be experimentally verified.

Alternatively, according to the second model language might influence perception at a later conceptual or decision stage rather than at the sensory stage, which is illustrated by the “feed-forward model.” To illustrate, in this model the motion word “rise” is processed in language-specific regions as well, where it activates its conceptual representation. The visual motion information is first processed in motion-sensitive area hMT+/V5 and subsequently up to a more conceptual level (“up"/"down”). Then, this conceptual representation of the visual stimulus interacts with the conceptual representation of the motion word. In this model, language modulates perception not by directly affecting the sensory processing stage in a top-down manner, but because visual information converges on the same conceptual representation as semantic information. This view is supported by recent neuroimaging evidence (Tan *et al*., 2008; Klemfuss *et al.*, 2012; Francken *et al.*, 2015): e.g. we have recently shown that the congruency of word-visual motion pairs (e.g. the word “rise” and upward visual motion) is reflected only in higher-order areas [left middle temporal gyrus (lMTG)], with activity in sensory visual areas (hMT+/V5) unchanged (Francken *et al*., 2015).

In sum, there is empirical support for both the feedback and the feed-forward model of language–perception interactions. Here we present an experimental procedure that allowed us to directly compare key predictions of the two models. Crucially, we manipulated the awareness level and thereby the nature of processing of the linguistic information by means of backward masking. Backward masking is a well-known experimental procedure to render briefly presented stimuli unaware by interspersing it with visual masks. Influential models of awareness in monkey electrophysiology (Lamme *et al.*, 2002) and human imaging studies (Del Cul *et al.*, 2007; Fahrenfort *et al.*, 2007) suggest that backward masking selectively disrupts feedback processing, while leaving feed-forward processing relatively intact (Lamme and Roelfsema, 2000; van Gaal and Lamme, 2012). Because backward masking selectively disrupts feedback processing, this experimental design allowed us to adjudicate between the feed-forward and feedback model of language–perception interactions. Here, masking disrupts the feedback, sometimes also referred to as “broadcasting” (Dehaene and Changeux, 2011), of the linguistic information from language areas in the left temporal lobe to sensory areas involved in word processing as well as sensory areas related to processing of the visual motion stimulus. Since we explore the effects of language on motion perception, we here refer to the latter type of feedback. Thus, the feedback model predicts that masking words will abolish the perceptual effect. In contrast, the feed-forward model predicts that masked words will still affect perception because these effects are supported by feed-forward processing to higher-level conceptual regions only.

Besides this main question, we had two additional questions. First, we were interested in the potential lateralization of (unaware) language–perception interactions. Previous studies have indicated that these interactions might be larger, or exclusively present, for visual information processed in the language-dominant left hemisphere (Gilbert *et al.*, 2006; Francken *et al*., 2015), although evidence is mixed (Witzel and Gegenfurtner, 2011). Therefore, we explored potential differences in lateralization effects between unmasked and masked words by presenting motion stimuli in the left and right hemifield.

Second, we wondered whether and how decision and control processes that evolve after the actual integration of perceptual and linguistic information might differ between unmasked and masked conditions. Since the motion words refer to upward and downward motion directions, control processes might become activated to suppress this information which might interfere with the motion discrimination task. Previous studies of response conflict (i.e. Stroop or flanker tasks) show that control mechanisms become activated with increasing response time (Ridderinkhof, 2002; Jiang *et al.*, 2013). However, at present, it is undecided whether these mechanisms are dependent on awareness of the (in)congruency of the stimulus or whether masked stimuli can evoke these conflict-control mechanisms as well (Kunde *et al.*, 2012).

## Materials and methods

### Participants

Thirty-eight healthy, right-handed participants with normal or corrected-to-normal vision (2 males, 36 females; age range: 18–29 years) took part in the two sessions of this experiment. All participants were native Dutch speakers and reported having no reading problems. The study was approved by the regional ethics committee and a written informed consent was obtained from the participants according to the Declaration of Helsinki. Compensation was 25 Euros, or course credit.

### Stimuli

Stimuli were generated using the Psychophysics Toolbox (Brainard, 1997) within MATLAB (MathWorks, Natick, MA, USA), and displayed on an ASUS LCD computer monitor (refresh rate 60 Hz, 1920 x 1080 resolution, size 50.9 x 28.6 cm). Stimuli were presented in white on a light-gray background. The visual random-dot motion (RDM) stimuli consisted of white dots (density = 2.5 dots/deg^2^; speed = 6.0°/s) plotted within a circular aperture (radius 7.5°). On every trial, the RDM stimulus was presented on either the left or right side of the screen (8.5° horizontal eccentricity from fixation to center of circular aperture) for 200 ms. In the first frame of the RDM stimulus, a random configuration of dots was presented within the annulus. Subsequently, on every frame a certain percentage of the dots was replotted consistently in one direction (upward or downward) on the next frame (see “Procedure”). Dots moving outside of the annulus and other remaining dots were replotted at a random location within the annulus.

Five verbs describing each direction of motion (in Dutch, here translated to English; upward: *grow, ascend, rise, climb, go up;* downward: *sink, descend, drop, dive, go down*), and 10 nonmotion verbs (*bet, mourn, exchange, glow, film, rest, cost, sweat, wish, relax*) were used in the experiment. Motions words and neutral words were matched for lexical frequency (taken from the CELEX database) and word length (5–8 letters) (both *P* > 0.2). Masks were randomly generated combinations of 10 consonant strings. Both words and masks were presented at the center of the screen, using capital letters in a mono-spaced font.

### Procedure

Participants performed a motion discrimination task (upward vs. downward motion) on a visual RDM stimulus (Fig. 1B). A central fixation cross (width 0.4 degrees) was presented throughout the trial, except when a word, mask or blank screen was presented. Each trial started with a centrally presented forward mask (50 ms) followed by a word (33 ms), which could either be a motion word or a nonmotion (neutral) word. Presentation of the words was pseudorandom within each block of the experiment. Awareness of the word was manipulated by presenting either backward masks (2 x 33 ms; unaware condition) or a blank screen (67 ms; aware condition) after word presentation. A short interstimulus interval (ISI) of 17 ms was always present after either of these screens. Next, a visual RDM stimulus was presented (200 ms) in either the left visual field (LVF) or in the right visual field (RVF). Participants had to indicate as quickly and accurately as possible whether the RDM contained upward or downward motion, while maintaining fixation at the central cross. The brief presentation time of the RDM stimulus served to minimize the chance of eye movements to the stimulus, as saccade latencies are in the order of ∼200 ms (Carpenter, 1988). Participants were instructed to respond as quickly and accurately as possible by pressing a keyboard button with either the index or middle finger of the right hand (counterbalanced across participants). The intertrial interval (ITI) was 1000–1166 ms.

In 10% of the trials, the motion discrimination task was followed by an additional task assessing the visibility of the words. Here, participants indicated whether the word presented earlier in the trial was a motion or a nonmotion word. These catch trials were included for two reasons. First, they ensured attention to the words, which enhances processing of the primes in both unmasked and masked conditions (Naccache *et al.*, 2002; Spruyt *et al.*, 2012). Second, catch trials were used to estimate word awareness. Participants were instructed to always respond to the catch question. They were explicitly told that there was a 50% chance of either motion or nonmotion words (10 different words of each category) in the catch trials. Note that nonmotion words were solely included to test for the visibility of the words.

The experiment consisted of two 1-h sessions on separate days within 1 week. In the first session, participants performed a training phase to familiarize them with the task and assess their individual motion coherence threshold at which they performed the motion discrimination task at 75% correct. Participants first practiced the motion discrimination task in three blocks with fixed coherence levels (80%, 55% and 30% respectively). The coherence level of the next training block was adjusted on the basis of performance in the previous blocks. The coherence level after the fourth training block was taken as the starting point for the Bayesian adaptive staircase procedure (Watson and Pelli, 1983), which was run separately for LVF and RVF stimuli. This was done to yield comparable task difficulty and performance in both visual fields and for all participants. The threshold for discrimination was defined as the percentage of coherent motion for which the staircase procedure predicted 75% accuracy. In both the remaining training blocks and the experiment, the coherence level was fixed within a block. The same Bayesian staircase procedure ran throughout the block; however, the actual coherence level was updated only between blocks (based on the estimate after the last trial of a block) to accommodate potential practice and fatigue effects over the course of the experiment. In the final training blocks, participants practiced the discrimination task while the words were presented and the catch task was added. During training, we provided participants with trial-by-trial feedback for both the motion task (except for the threshold estimation block) and the catch trial task by means of a green or red fixation cross for correct and incorrect responses, respectively. The training was followed by a practice phase, in which participants completed 440 trials (5 blocks of 88 trials) of the actual experimental task to familiarize them with the task and to avoid practice effects in the actual experimental blocks. On the second day, participants first completed a short training (88 trials). The experiment on the second day consisted of 10 blocks of 80 trials (800 trials in total). All analyses reported here are based on the 10 experimental blocks in this final session. Summary feedback (percentage correct) was provided during the break after each block.

One participant was excluded because performance on the unmasked trials of the motion discrimination task was <60% correct. Therefore, analyses were performed on 37 participants.

### Statistical analysis

We calculated congruency effects for reaction times (RT) on correct trials and error rates (ER). On congruent trials, the motion described by the word matched the direction of visual motion, e.g. “rise” followed by a stimulus with upward moving dots. On incongruent trials, the motion described by the word and the direction of visual motion did not match. Missed trials and trials with RTs that were >3 SD than the individual subject mean RT were excluded from the analyses (in total 2.3%). Each of the two behavioral measures was subjected to a repeated measures analysis of variance (ANOVA), including factors “Congruency” (congruent, incongruent), “Awareness” (aware, unaware) and “Visual Field” (LVF, RVF).

To further assess the potentially different effects of unmasked and masked words on motion perception, we used Bayesian Statistics (Jeffreys, 1961; Rouder *et al.*, 2009) and delta plots (Ridderinkhof, 2002). Previous studies have indicated that language–perception interactions might be larger, or exclusively present, for visual information processed in the language-dominant left hemisphere (Gilbert *et al*., 2006; Francken *et al*., 2015), while others have failed to replicate these effects (Witzel and Gegenfurtner, 2011). To differentiate between the presence and absence of evidence for the null hypothesis (no lateralization of congruency effects), we calculated Bayes Factors (BFs). BFs express evidence ratios between hypotheses, and therefore provide direct information about the relative likelihood of the alternative vs. the null hypothesis. A BF of ∼1 indicates no preference for either the null or the alternative hypothesis, and in large samples BFs will converge to either 0 or infinity when the null or alternative hypothesis is true respectively (Rouder *et al*., 2009). By convention, a BF likelihood ratio of >3/1 provides moderate evidence for the alternative hypothesis, >10/1 provides strong evidence for the alternative hypothesis and >30/1 provides very strong evidence for the alternative hypothesis (Jeffreys, 1961). Equivalently, a BF of <1/3 provides moderate support for the null hypothesis, <1/10 provides strong support for the null hypothesis and <1/30 provides very strong support for the null hypothesis. BF ratios between 1/3 and 3/1 provide no evidence for either the null or alternative hypothesis.

Second, we wondered whether decision and control processes might become activated with increasing response time to suppress the interference of the task-irrelevant linguistic information with the motion discrimination task. Therefore, we calculated delta plots (reflecting the RT congruency effects for different RT bins) and conditional accuracy functions (reflecting the ER congruency effects for different RT bins) to assess the congruency effects across the response time distribution. For each visibility condition, every participant’s trials (correct trials only) were sorted on RT and subsequently equally divided over 10 RT bins (separate bins for congruent and incongruent trials). Next we performed a repeated measures ANOVA including factors “Congruency” (congruent, incongruent), “Awareness” (aware, unaware) and “RT bin” (1 to 10). Previous studies show that the build-up of suppression of interference is maximal at the slowest RT bins (Ridderinkhof, 2002; Forstmann *et al*., 2008; Jiang *et al*., 2013). Therefore, we performed additional planned paired *t*-tests on RTs between the first (second minus first RT bin) and last (tenth minus ninth RT bin) slopes to assess whether conflict control became stronger over response time in the current study as well. The strength of automatic response activation by the motion words is inferred from the pattern of errors present at the fastest RT bins. Stronger response capture is associated with a higher percentage of fast errors (Ridderinkhof, 2002). Thus, the critical measure for conflict control effects on ERs was the presence of a three-way interaction between congruency, awareness and RT bin.

To assess the awareness of the words, we calculated the accuracy and *d’* in the catch trials. Percentage correct was defined as the percentage of trials on which participants correctly indicated whether the word was a motion word or not. *d'* is an unbiased measure of the discriminability sensitivity of the observer (Macmillan and Creelman, 2005). *d’* for the unmasked and masked conditions were first compared to each other using paired *t*-tests and subsequently compared with zero using one-sample *t*-tests. Following this, we used the accuracy in binomial tests to determine for every participant whether the performance was above chance (50% correct). In addition, we calculated correlations between *d’* and congruency effects. We used a regression approach, referred to as Greenwald’s method (Greenwald *et al.*, 1995) to test whether the reported congruency effects were still significant when discrimination performance was extrapolated to zero visibility (*d*’ = 0) [see Greenwald *et al*. (1995) and Hannula *et al*. (2005) for further discussion and justification of this method]. Finally, we split the participants into a low visibility (*d*' < median) and a high visibility (*d*' > median) group and we performed an ANOVA across the masked conditions with the factors Congruency (2) and Group (2) to test for potential differences between the congruency effects (CE) of the low and high visibility groups.

## Results

### Word discriminability

We excluded 4 of 37 participants whose discrimination performance of the masked words was above chance level (binominal test, *P* < 0.1), because for these four participants we could not be sure that they were unable to discriminate the masked words. On a group level (for the remaining 33 participants), the discriminability of the words (motion vs. no motion) was markedly lower when the words were masked (unaware condition) than when they were not masked (aware condition) [difference *t*(32) = 10.22, *P* < 0.001; unaware *d'* = 0.16, corresponding to 52.8% correct responses, *t*(32) = 2.57, *P* = 0.015; aware *d'* = 2.25, 84.0% correct, *t*(32) = 11.11, *P* < 0.001]. To assess whether residual visibility of the masked motion words is responsible for any of the effects on visual motion perception, we performed several control analyses (see below).

### Do masked motion words affect visual motion perception?

**Figure 2:**
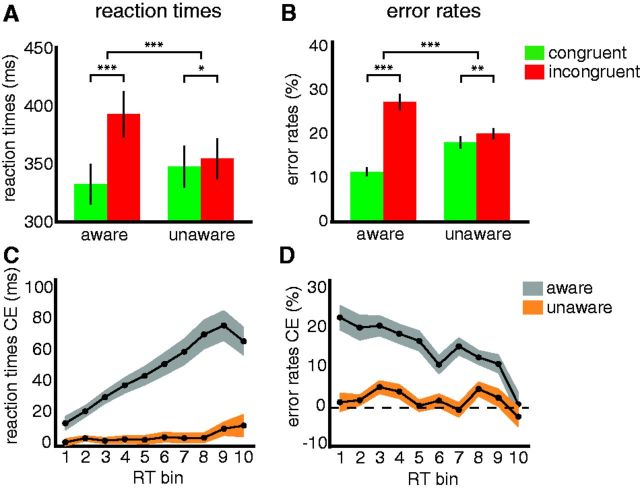
Results (**A**) Mean reaction times (in ms) in the unmasked (aware, left bars) and masked (unaware, right bars) conditions for visual motion stimuli that were preceded by a congruent (green) motion word were faster than when preceded by an incongruent (red) motion word. (**B**) Mean error rates (%) in the aware condition and unaware condition were lower for congruent than incongruent motion words. (**C**) The delta plot for reaction times (in ms) congruency effects (incongruent-congruent, CE) in the conscious (gray) condition showed the typical RT conflict-control profile with an initial CE increase over RT bins and a CE decrease in the last bin. In the unaware condition (orange), the CE was not affected by response time and did not decrease in the last bin. (**D**) Conditional accuracy functions for error rates (%) CE. Stronger response capture is associated with a higher percentage of fast errors. This pattern of decreasing CE across RT bins is present for the aware condition, but not for the unaware condition. Error bars denote SEM. *P < 0.05, **P < 0.01, ***P < 0.001.

We first focus on the effects of word awareness on word-motion congruency. Participants responded faster to the motion stimuli when they were preceded by a congruent motion word than by an incongruent motion word (main effect of congruency: *F*_1,32_ = 80.11, *P* < 0.001). This congruency effect was modulated by word awareness (congruency x awareness: *F*_1,32_ = 46.20, *p* < 0.001), indicating that the difference between congruent and incongruent conditions was larger when the words were unmasked than when they were masked. Crucially, however, the congruency effect was present both when the words were unmasked (congruent: RT = 335 ms; incongruent: RT = 395 ms; ΔRT = 60 ms, *F*_1,32_ = 69.60, *P* < 0.001; Fig. 2A), and when they were masked (congruent: RT = 350 ms; incongruent: RT = 356 ms; ΔRT = 6 ms, *F*_1,32_ = 6.77, *P* = 0.014).

The congruency effects in error rates go in the same direction for both unmasked and masked words. On average, participants answered 81.3% of trials correctly (±6.7%, mean ± SD) at an average motion coherence level of 48.1% for the LVF (±18.3%, mean ± SD) and 46.5% for the RVF (±16.3%, mean ± SD). Participants made fewer errors for congruent compared to incongruent trials (main effect of congruency: *F*_1,32_ = 130.19, *P* < 0.001; aware: congruent: 11.3%; incongruent: 26.9%; ΔER = 15.6%, *F*_1,32_ = 126.34, *P* < 0.001; unaware: congruent: 17.8%; incongruent: 19.9%; ΔER = 2.1%, *F*_1,32_ = 10.89, *P* = 0.002; Fig. 2B). Again, the congruency effect was larger for unmasked words than for masked words (congruency x awareness *F*_1,32_ = 81.30, *P* < 0.001).

The word discrimination trials, interspersed throughout the experiment, revealed that performance (visibility) was low for masked words (52.8% correct responses). However, on a group level, visibility was higher than chance level. Therefore, to check whether incidental word visibility might have been responsible for the observed congruency effects, we performed several control analyses. First, if incidental word visibility were responsible for the observed congruency effects, one would expect reliable positive correlations between discrimination scores (*d’*) and congruency effects. However, this was not the case for RTs (r_s_ = 0.17, *P* = 0.34) and ERs (r_s_ = 0.19, *P* = 0.28). Further, we used a regression approach, referred to as Greenwald’s method (Greenwald *et al*., 1995) to test whether the reported congruency effects were still significant when discrimination performance was extrapolated to zero visibility (*d’* = 0). Indeed, the linear regression analyses revealed a significant intercept for ERs (intercept = 1.73, *P* = 0.018) and a trend for RTs (intercept = 5.42, *P* = 0.069) which, although not conclusive, further suggests that congruency effects were induced by masked words that could not be perceived consciously. Next, we split the participants (*n* = 33) into a low visibility (d' < median, *n* = 16) and a high visibility (d' > median, *n* = 17) group. An ANOVA across the masked conditions indicated that there were no significant differences between the congruency effects (CE) between the low and high visibility groups (ERs: low visibility group CE = 1.7%; high visibility group CE = 2.4%; *F_1,31_* = 0.29, *P* = 0.59; BF: 1/3.5; RTs: low visibility group CE = 3 ms; high visibility group CE = 10 ms; *F_1,31_* = 1.52, *P* = 0.23; BF: 1/2.1). Below, we will describe qualitative differences between the masked and unmasked condition that further suggest that subjects were unable to perceive the masked words (Jacoby, 1991; Merikle *et al.*, 2001).

In summary, both RTs and ERs showed that masked words influenced motion perception, although to a lesser extent than words that were not masked. These results are in line with feed-forward models explaining the effects of language on perception, but not with feedback models.

### Spatial characteristics of language–perception interactions

We reasoned that masked and unmasked words might influence perception in qualitatively different ways, due to the different neural processes involved in both situations (feed-forward vs. recurrent/feedback processing, respectively). First, we tested whether the congruency effect for masked and unmasked conditions was differentially modulated by the visual field in which the visual motion stimuli were presented. For both RTs and ERs, there was no interaction between congruency, awareness and visual field (both Ps > 0.7; RTs: aware: ΔRT LVF: 56 ms, ΔRT RVF: 62 ms; unaware: ΔRT LVF: 5 ms, ΔRT RVF: 8 ms; ERs: aware: ΔER LVF: 15.5%, ΔER RVF: 15.7%; unaware: ΔER LVF: 2.1%, ΔER RVF: 2.1%). Frequentist statistics provide a measure of confidence in rejecting the null hypothesis, but not a measure of confidence in the null hypothesis itself. In order to verify the true absence of lateralized effects, we calculated BFs separately for both masking conditions. We observed moderate evidence (BF < 1/3) for the null hypothesis, indicating no effects of visual field on congruency for both masked and unmasked conditions, both in terms of RTs and ERs (aware: RTs: BF = 1/4.5; ERs: BF = 1/7.2; unaware: RTs: BF = 1/6.2; ERs: BF = 1/7.4).

### Temporal characteristics of language–perception interactions

Our previous analyses suggest that language influences perception at higher-level conceptual or decision stages (in a feed-forward manner) rather than at low-level sensory stages (in a feedback manner). Therefore, in follow-up analyses, we explored the possible differences in decision and control processes that evolve after the actual integration of perceptual and linguistic information for masked and unmasked conditions. To do so, we calculated the so-called “delta plots,” reflecting the RT congruency effects for different RT bins and “conditional accuracy functions,” reflecting the ER congruency effects for different RT bins, to assess the congruency effects across the response time distribution (Ridderinkhof, 2002; Forstmann *et al*., 2008; Jiang *et al*., 2013). The delta plots for the unmasked condition showed the typical RT conflict-control profile (Ridderinkhof, 2002; Forstmann *et al*., 2008; Jiang *et al*., 2013). The congruency effects first increased over response time (congruency x RT bin: *F*_1,32_ = 24.47, *P* < 0.001) and later decreased in the last bin (2nd–1st vs. 10th–9th bin: *T*_32_ = 2.35, *P* = 0.025; Fig. 2C). There was a significant difference between the congruency effects for masked and unmasked conditions over response time (congruency x awareness x RT bin: *F*_1,32_ = 13.08, *P* < 0.001) driven by the overall increase in the congruency effect for unmasked but not for masked words. Interestingly, for masked words, the congruency effect was not affected by response time (congruency x RT bin: *F*_1,32_ = 1.39, *P* = 0.19) and the RT delta plot did not show the typical control-related decrease in the congruency effect for the last RT bin (2nd–1st vs. 10th–9th bin: *T*_32_ = −0.06, *P* = 0.955; difference between aware and unaware conditions: *T*_32_ = 1.47, *P* = 0.15). Thus, for the unmasked condition, we found a quick increase in the RT congruency effect with response time, followed by a later decrease, probably as a consequence of the activation of interference control mechanisms. In the masked condition, however, RT congruency effects were stable over RT bins and did not show any of the control-dynamics as observed in the unmasked condition. The strength of automatic response activation by the motion words is inferred from the pattern of errors present at the shortest RT bins. Stronger response capture is associated with a higher percentage of fast errors (Ridderinkhof, 2002). Again, for accuracy there was a significant three-way interaction (congruency x awareness x RT bin: *F*_1,32_ = 3.54, *P* < 0.001), indicating differential effects of response time on congruency for the unmasked and masked conditions. For the unmasked condition, the congruency effect decreased over response time (congruency x RT bin: *F*_1,32_ = 7.59, *P* < 0.001), thus showing the typical pattern of these conditional accuracy functions. In contrast, the masked condition showed no modulation over RT bins (congruency x RT bin: *F*_1,32_ = 1.35, *P* = 0.22; Fig. 2D). Thus, only for the unmasked words a large ER congruency effect was present for the fast RT bins, which is in line with the fact that voluntary control mechanisms take time to be initiated. In the masked condition, however, the data pattern was very different and again, like for RT, did not show any of these control-dynamics. Note that this last set of analyses also further suggests that the masked words were invisible. We observed qualitative differences in the effects of masked vs. unmasked motion words on voluntary control mechanisms, but similar effects on congruency effects (reflecting language–perception interactions). These qualitative differences are generally considered as convincing evidence for unconscious perception (Jacoby, 1991; Merikle *et al*., 2001).

In sum, when the word and the motion stimulus were congruent, both unmasked and masked words sped up motion discrimination and increased discrimination performance compared to incongruent word–motion pairs. Language–perception interactions were equally present for visual stimuli presented in the left and right hemifield, but only in the unmasked condition were voluntary control mechanisms activated across response time to reduce linguistic interference.

## Discussion

We investigated whether language affects perception in a feed-forward or a feedback manner by disrupting the processing of motion words by means of backward masking. The rationale behind this experimental design is that feedback processing is disrupted by masking, as revealed by empirical evidence from monkey electrophysiology and human neuroimaging studies (Lamme and Roelfsema, 2000; Lamme *et al*., 2002; Del Cul *et al*., 2007; Fahrenfort *et al*., 2007). Hence, the feedback model predicts that interactions between language and perception will be abolished under masked conditions. Since backward masking does not affect feed-forward processing, the feed-forward model predicts that effects of language on perception will still be present when the words are masked. Our results support the feed-forward model: when motion words were masked, motion words that were congruent with the direction of the visual motion stimulus resulted in faster and more accurate visual motion direction discrimination relative to incongruent conditions. Thus, our results suggest that language changes perception at a higher, conceptual level, rather than at the lower, sensory level. With several control analyses, we verified that our results are unlikely driven by residual visibility of the masked motion words.

A number of previous studies are in line with this interpretation. We recently found that congruent word–motion pairs elicit higher BOLD activity than incongruent combinations in the left middle temporal gyrus (Francken *et al*., 2015), an area involved in both lexical retrieval and semantic integration (Hagoort *et al.*, 2009; Menenti *et al.*, 2011). Crucially, there were no effects in motion-sensitive visual areas such as hMT+/V5. Interestingly, support for this effect was also found by an fMRI study in which linguistic material was only implicitly included. Tan *et al.* (2008) had participants judge whether two colored squares had the same or a different color. Even though the linguistic color vocabulary was irrelevant for the perceptual discrimination task, left temporo-parietal circuits associated with word-finding processes were activated more strongly when subjects had to discriminate between hard-to-name colors compared to easy-to-name colors. These studies indicate that the interaction between language and perception is mediated by “language areas” that integrate linguistic and visual information. These data stand in sharp contrast to previous proposals that linguistic material describing motion elicits a “perceptual simulation” in low-level visual areas similar to actually seeing motion (Saygin *et al*., 2010).

How does language change perceptual decision making according to the feed-forward model? We reason that visual motion stimuli might be conceptually categorized as reflecting evidence for “upward” and “downward” motion directions, since participants are required to make a categorical perceptual decision. This may cause conceptual representations to be automatically activated (Tan *et al*., 2008; Thierry *et al*., 2009), even though they are not required for task performance. If the conceptual representation activated by the visual motion stimulus matches the conceptual representation that is activated by the motion word, this then results in more activity in lMTG (Francken *et al*., 2015), as well as improved behavioral performance. It is also possible that language–perception interactions take place at an even later decision stage. Taken together, the reason why masked (unaware) words are still able to change perception according to the feed-forward model is that the interaction does not depend on feedback of linguistic information to sensory areas. The only requirement is that the masked words are semantically processed, which does indeed occur despite backward masking (Kouider and Dehaene, 2007; van Gaal and Lamme, 2012). Although we show that feed-forward processing is sufficient for language–perception interactions to occur, our data cannot adjudicate whether larger congruency effects under unmasked conditions are due to additional feedback processing or increased stimulus strength.

Proponents of the feedback model argue that language–perception interactions might be dependent on visual “mental imagery,” which is the conscious, internal generation of images (Kosslyn *et al.*, 2001). This process would require feedback from regions up in the cortical hierarchy together with language areas in order to affect low-level sensory processing. For example, when reading stories describing motion events, participants showed a motion aftereffect illusion, which can be interpreted as evidence for direction-selective motion adaptation in the visual system (Dils and Boroditsky, 2010). Interestingly, individuals differed in how early in the story the effect appeared, and this difference was predicted by the strength of an individual’s motion aftereffect following explicit motion imagery. Thus, when imagery is sufficiently vivid, language appears to induce changes in the visual system. However, by showing that masked words can still influence perception, we demonstrate here that mental imagery cannot account for all instances of linguistic modulations of perception.

We further qualified the spatial and temporal characteristics of the language–perception interactions. First of all, we did not observe any lateralization of the reported effects. Interestingly, in our previous study in which words were unattended, but not masked, the RT effect (but not the accuracy effect) was lateralized to the right visual field (Francken *et al*., 2015). Therefore, our findings provide an alternative explanation for the often reported (and debated) observation that language exerts stronger effects for stimuli presented in the right visual field (Gilbert *et al*., 2006; Regier and Kay, 2009; Witzel and Gegenfurtner, 2011). This lateralization is explained by the fact that information from the right visual field would have preferential access to the left-lateralized language system (Gilbert *et al*., 2006; Regier and Kay, 2009; Klemfuss *et al*., 2012). Although this is an intuitively appealing idea, our data suggest that this depends on the degree to which the linguistic information is attended: unattended stimuli might show lateralized effects, whereas attended stimuli might not. Future studies are clearly needed to further explore this hypothesis in more detail.

Finally, we observed that decision and control processes that evolve after the integration of perceptual and linguistic information differed between unmasked and masked conditions. In line with previous studies of response conflict (Ridderinkhof, 2002), we reasoned that to suppress the interference of the task-irrelevant words, inhibitory control mechanisms might be activated with increasing response times. Interestingly, these control dynamics were uniquely observed for unmasked words. Thus, although masked words have the power to change perceptual decisions about motion direction, late voluntary control mechanisms to suppress the irrelevant linguistic information were not activated (Tsushima *et al.*, 2006). First, this finding provides evidence for the notion that there was a clear qualitative difference in awareness between the masked and unmasked conditions. Second, these results inform recent discussions about the role of consciousness in cognitive control and the potential control processes that can unconscious stimuli might be able to affect or even initiate (Kunde *et al*., 2012; van Gaal *et al.*, 2012; Ansorge *et al.*, 2014). Previously, it has been shown that masked stimuli that “explicitly” signal the need for control (e.g. an unconscious stop-signal or an unconscious task-switching cue) can elicit behavioral and neural indices of control behavior (Lau and Passingham, 2007; van Gaal *et al.*, 2008, 2009, 2010). However, it has recently been argued that “implicit” cues, such as specific task properties that have to be derived from repeated exposure to the trials, might not (Kunde *et al*., 2012). Because in the present experiment masked words were not explicitly associated with control processes, and are in fact irrelevant to perform the motion discrimination task, this might be a situation in which control processes are dependent on awareness. However, some previous studies using nonverbal material (i.e. arrow stimuli) in typical priming tasks have observed control mechanisms irrespective of conflict awareness (van Gaal *et al*., 2010; Francken *et al.*, 2011; Desender *et al.*, 2013), although evidence is mixed (Kunde, 2003; Ansorge *et al.*, 2011) (for reviews see Ansorge *et al*., 2014; Kunde *et al*., 2012). It might be that with the current set-up initial conflict was too small to initiate further control operations in the masked condition (Kunde *et al*., 2012). Future studies should be performed to further explore in which situations control operations can be triggered implicitly (and explicitly) and in which situations it cannot, and what factors underlie these differences.

In conclusion, here we have manipulated word awareness in a visual motion discrimination task to explore at what level of processing the influence of language on perception takes place. Specifically, we were able to dissociate feed-forward models from feedback models of language–perception interactions. We observed a clear influence of language on motion discrimination for both masked (unaware) and unmasked (aware) words. Because feed-forward processing remains intact whereas feedback to low-level sensory areas is disrupted by masking, these results can only be explained by a feed-forward model. Therefore, these findings provide evidence for the hypothesis that language–perception interactions occur at stages beyond low-level sensory regions and are mainly driven by interactions at higher-level conceptual and decision stages.

## Author contributions

J.C.F., E.L.M., P.H., S.vG. and F.P.deL. developed the study concept and design. Data collection was performed by O.M.R. J.C.F., O.M.R. and E.L.M. performed the data analysis and interpretation under the supervision of F.P.deL. and S.vG. J.C.F and S.vG. drafted the manuscript, and F.P.deL. and P.H. provided critical revisions. All authors approved the final version of the manuscript for submission.

## Data Availability

Data is available on request. *Conflict of interest statement*. None declared.
